# The Association Between Internet Gaming Disorder and Sensation Seeking Among Arab Adolescents

**DOI:** 10.3389/fpsyt.2022.905553

**Published:** 2022-07-15

**Authors:** Mohamed S. Hamid, Eid Abo Hamza, Zaheer Hussain, Aisha AlAhmadi

**Affiliations:** ^1^Faculty of Education, Ain Shams University, Cairo, Egypt; ^2^College of Humanities and Sciences, Ajman University, Ajman, United Arab Emirates; ^3^Faculty of Education, Tanta University, Tanta, Egypt; ^4^School of Social Sciences, Nottingham Trent University, Nottingham, United Kingdom; ^5^Department of Psychological Sciences, Qatar University, Doha, Qatar

**Keywords:** internet gaming disorder, sensation seeking, addiction, online gaming, Arab adolescents

## Abstract

Research on internet gaming disorder (IGD) has increased considerably over the last decade. Although most IGD research has focused on Western or South Asian samples, it is critical to understand and assess this disorder among other populations. The present study investigated the association between IGD and sensation seeking among Arab adolescents. An online survey consisting of the short internet gaming disorder short scale (IGDSS) and brief sensation seeking scale (B-SSS) was completed by 260 participants (mean age = 14.61, *SD* = 2.43). The results showed that sensation seeking behaviours were associated with higher gaming hours and greater chances of exhibiting IGD. The findings support the current perspective of understanding this disorder from a disease framework as it highlights the relevance of behavioural components in gaming addiction. Importantly, the results will further aid the development of reliable diagnosis and efficacious treatments within clinical practices.

## Introduction

Online gaming is a popular leisure activity for millions of people around the world because it provides an environment where individuals can form new relationships, adopt new online personas and escape from reality (1). Gaming can be a beneficial activity as it improves integration of perceptual information into the brain and hand-eye coordination (2). However, for some it may lead to problematic use as excessive gaming can result in a loss of impulse control, behavioural inhibition, executive functioning and attention (1, 3). As the internet has evolved and become a dominant part of life, problems associated with internet use have increased. Some researchers have suggested that gaming behaviour that is characterised as being excessive, maladaptive, and repetitive relates very closely to substance addiction (4, 5) and can therefore be characterised as a behavioural addiction. The term behavioural addictions is now recognised by many researchers. Various types of behavioural addictions have been studied, including internet addiction (IA) (6), smartphone addiction (7, 8), social networking addiction (9), and pathological gambling (10). Researchers have argued that all of these online activities can be addictive and can negatively affect the user’s mental health (11–13). Despite there being consensus on the criteria of maladaptive online gaming, the aetiology of this addiction has yet to be studied in detail. Personality traits such as neuroticism, aggression, hostility and sensation seeking have been suggested as risk factors (14).

Several terms have been used to describe problematic gaming behaviour. These include internet gaming disorder (IGD), computer game addiction, video game addiction, game overuse, and pathological internet use. More recent research studies have used the term IGD (15, 16). It is important to note that IGD is not yet considered a clinical disorder in the fifth edition of the Diagnostic and Statistical Manual of Mental Disorders [DSM-5; (17)], further research into the behaviour was encouraged. The DSM-5 clearly defined symptom criteria for IGD, one must display five or more diagnostic criteria during a 12 month period. The criteria include evidence of (1) preoccupation with games, (2) withdrawal symptoms, (3) the need to spend increasing amounts of time engaged with games (tolerance), (4) unsuccessful attempts to control or reduce game play, (5) loss of interest in real-life relationships, hobbies, and other entertainment, (6) continued excessive use of games despite knowledge of psychosocial problems, (7) deception of others regarding amount of gaming, (8) use of games to relieve a negative mood, and (9) jeopardising or losing a significant relationship, job, or educational opportunity because of gaming. This criterion has been used increasingly by researchers to investigate problematic gaming behaviour.

As research continues to be conducted into problematic behaviours, concerns about the impact of IGD on the lives of gamers has led to an increase in research studies that highlight potential negative effects. Ohayon et al. (18) investigated IGD among United States university students. The results showed that IGD was prevalent in 5.3% of the sample, IGD was associated with non-restorative sleep, excessive daytime fatigue, depressive mood, and social anxiety disorder. A range of other predictors of IGD have been reported in research, these include procrastination (19), alexithymia, depression, and anxiety (20, 21), physical and psychological harm (22), and emotional problems (23). Some studies have investigated variables that may be seen as risk factors and that may predict IGD. For example, Rho et al. (24) reported that the risk factors of functional and dysfunctional impulsivity, self-control, anxiety, pursuit of desired appetitive goals, money spent on gaming, weekday game time, offline community meeting attendance, and game community membership were significantly associated with IGD. Müller et al. (25) reported that higher neuroticism, decreased conscientiousness, and low extraversion were associated with IGD. Fumero et al. (26) found that anxiety, time spent playing predicted IGD, social skills and family functioning indirectly predicted IGD. The researchers also found that a different set of variables was associated with IGD depending on gender; lower family functioning was associated with IGD in females, hostility and social skills deficit was associated with IGD in males. One study reported that impulsiveness and high amounts of time gaming may be risk factors for IGD (27). Altogether, research studies have shown that there are numerous variables or risk factors associated with IGD.

There are several theories that have proven useful in understanding the mechanisms underlying the phenomenon of IGD. The cognitive-behavioural model of IGD (28, 29) focuses on the role of three domains in addictive behaviour; (1) motivational drives related to reward-seeking and stress-reduction, (2) behavioural control relating to executive inhibition, and (3) decision-making that weighs the consequences of engaging in motivated behaviours. The Compensatory internet use model (30) proposes that conclusion can be drawn by understanding motivations and how they mediate the relationship between psychosocial wellbeing and problematic internet use. Recently, the interaction of person-affect-cognition-execution (I-PACE) model (31) describes how the interaction between predisposing variables (e.g., psychopathological variables, interpersonal sensitivity) and cognitive and affective mechanisms could result in the loss of control and then consequently lead to IGD. Taken together, theories of addictive behaviour have proven useful in explaining and understanding IGD and related behaviours.

Given the theoretical approaches and the discussion of predisposing variables, one variable that has been discussed and associated with high risk and addictive behaviours is sensation seeking [SS; (32–34)]. Research studies have reported associations between sensation seeking and severity of internet risk (35): Floros et al. (36) reported elevated SS among IA treatment seekers, Nejadfard and Hosseinsabet (37) reported significant associations between SS and IA. However, contrary to these research findings, inconsistent results have also been reported; Müller et al. (38) found decreased SS in patients with IA, a meta-analysis concluded that there were no signs of higher SS in gambling disorder [GD; (39)], also, no relationships between GD and SS have been reported (40). High sensation seekers prefer sensations that are varied, novel, and complex (41). Online games include all of these features and game companies are always developing new versions of games that contain advanced graphics and rich audio and visual effects. Research findings are inconsistent on the issue of sensation seeking, further examination is required.

Previous research has examined IGD among samples in western and Asian cultures identifying problems with dealing with Internet use (42). There is a lack of research literature that investigates IGD amongst the Arab population, studying this behaviour among this culture would provide unique and interesting insights. The present study sought to further understand IGD and sensation seeking using an online survey method. The focus of the study was on Arab adolescents, this is important because internet penetration rates have greatly increased among populations from the Middle East and North Africa (43). Little is known about the make-up of gamers from Arab countries, therefore an exploration of gaming, IGD, and variables that are associated with gaming is warranted. Previous IGD research among non-Arab gamers have reported interesting findings in regards to gender, education level, and gaming time (44–47). Little is known about these variables and how they may be associated with IGD among Arab gamers. The aims of the current study were to examine IGD and its potential associations with sensation seeking, gender, time spent gaming, and education level among a sample of Arab adolescents. IGD prevalence rates were also examined.

## Materials and Methods

### Participants

There were 260 participants in this study that consisted of 56.2% females ranging between the ages of 8 and 18, (*Mean age* = 14.61, *SD* = 2.43). Most participants were in high school (61.2%), followed by elementary level (29.2%), and finally prep school (9.5%). It was found that most of the participants (44.6%) played video games up to an hour per day, while 21.6% played between 1 and 3 h a day, 14.6% played between 3 and 6 h per day, while the remaining 19.2% played 6 h or more a day. Table 1 shows the participants’ characteristics.

**TABLE 1:** Descriptive statistic for participants.

Gender					

		Frequency	Percent	Valid percent	Cumulative percent
	Male	114	43.8	43.8	43.8
	Female	146	56.2	56.2	100.0
	Total	260	100.0	100.0	

**Age**					

	***N***	**Minimum**	**Maximum**	**Mean**	**Std. deviation**

Age	259	8.00	18.00	14.60	2.43
	259				

**Academic level**					

		**Frequency**	**Percent**	**Valid percent**	**Cumulative percent**

	Elementary	76	29.2	29.2	29.2
	Prep school	25	9.6	9.6	38.8
	High school	159	61.2	61.2	100.0
	Total	260	100.0	100.0	

**Gaming hours**					

	***N***	**Minimum**	**Maximum**	**Mean**	**Std. deviation**

Gaming hours	259	1.00	5.00	2.18	1.46

### Ethics

The study was approved by the ethics committee of the research team’s university. The study was carried out in accordance with the Declaration of Helsinki. All participants were informed about the study and all provided informed consent.

### Design and Materials

A cross-sectional online survey design was utilised in the present study. An online survey was developed with the use of *Qualtrics* Survey Software. The survey comprised of demographic questions and questions regarding the participants’ education level. The survey also comprised of a measure online gaming time and psychological instruments to assess the associations between IGD severity and sensation seeking, these instruments are described below.

#### The Internet Gaming Disorder Short Scale

**FIGURE 1:**
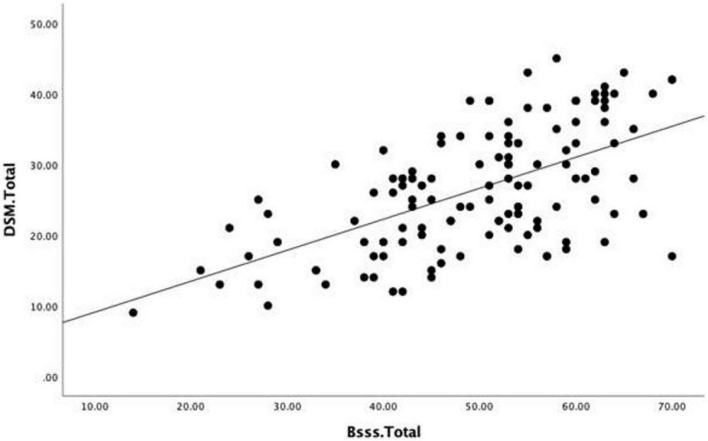
Proportion of total BSS and DSM scores identified by participants (*n* = 257).

The Internet Gaming Disorder Short Scale [IGDSS; (48)] is a tool used to assess the severity of the diagnostic and statistical manual of mental disorders (DSM) symptoms in online gamers over 12 months. The scale consists of 9 items answered on a 5-point Likert scale. The total score of items range from 9 to 45, with higher scores indicating greater severity in IGD symptomology (see Figure 1). The IGDSS has good psychometric properties in Arab contexts (α = 0.856) which was supported with exploratory and confirmatory Factor Analysis.

#### The Brief Sensation Seeking Scale

The Brief Sensation Seeking Scale [B-SSS; (48)] is adapted from the Form V of the Sensation Seeking Scale [SSS-V; (49)] and tailored for Arab speaking adolescents. It has been validated in Arab populations with good psychometric properties (α = 0.882). The Arabic version consist of 14 items answered on a five-point Likert scale (strongly agree to strongly disagree) and based on four factors: thrill and adventure seeking, disinhibition, experience seeking, and boredom susceptibility.

#### Time Spent Online Gaming

Time spent online gaming was measured by asking participants to state how many minutes they spent online gaming each day.

### Procedure

A recruitment message was posted online and sent *via* email to students inviting them to participate in the study. The recruitment message included information about the purpose of the study. When participants began the survey, they were presented with a participant information page followed by clear instructions on how to complete the survey. All participants were assured that their data would remain anonymous and confidential. A debriefing statement at the end of the survey reiterated the purpose of the study and informed participants of their right to withdraw from the study.

### Statistical Analysis

Descriptive statistics for the scales (mean scores, standard deviations, Cronbach’s alpha), Pearson’s correlation coefficients, linear regression, *t*-tests, and multivariate analysis of variance (MANOVA) were calculated using IBM SPSS for Windows, version 26.0. The significance limit was set at *p* < 0.05.

## Results

A bivariate correlation was conducted to evaluate whether higher scores on the Sensation Seeking Scale (B-SSS) would correlate with higher scores on the Internet Gaming Disorder (IGDSS). To assess the size and direction of the linear relationship between both scores, a bivariate Pearson’s product-moment correlation coefficient (*r*) was calculated. The bivariate correlation was positive and strong, *r*(257) = 0.587, *p* < 0.001. A calculation of *r*^2^ showed that 34.5% of variance in participants’ IGD scores can be explained by the variability in SS scores (see Table 2).

**TABLE 2:** Summary of correlations between Zscore BSS.Total and Zscore DSM.Total.

Correlations			
		Zscore (BSS.Total)	Zscore (DSM.Total)
Zscore (BSS.Total)	Pearson correlation	1	0.587**
	Sig. (2-tailed)		<0.001
	*N*	259	259
Zscore (DSM.Total)	Pearson correlation	0.587**	1
	Sig. (2-tailed)	<0.001	
	*N*	259	259

**Correlation is significant at 0.01 level (2-tailed).

The linear regression equation for predicting the IGD is


Predicted⁢Internet⁢Gaming⁢Disorder=4.69+0.44

Sensation⁢Seeking

This equation indicates that the relationship between both variables was linear and heteroscedastic. In addition, for every unit increase in SS, it was predicted that IGD would increase by 0.44.

An independent samples *t* test was carried out to compare average SS scores reported by male participants (*n* = 114) to the average SS scores reported by females (*n* = 145). Levene’s test was not significant (*p* = 0.749), therefore equal variance was assumed. The *t* test was statistically significant, with the male cohort reporting (*M* = 51.65, *SD* = 11.60) on average 3.06 units higher on SS, 95% CI [0.277, 5.85], than female participants (*M* = 48.58, *SD* = 11.06), *t*(257) = 2.165, *p* = 0.031, *d* = 0.27 (see Table 3).

**TABLE 3:** Means, standard deviations, and *t*-test for equality of means of sensation-seeking (SS) according to gender.

	Gender	*n*	*M*	*SD*	*t*	*df*	Sig.
Sensation-seeking	Male	114	51.65	11.60	2.17	257	0.031
	Female	145	48.59	11.06			

**FIGURE 2:**
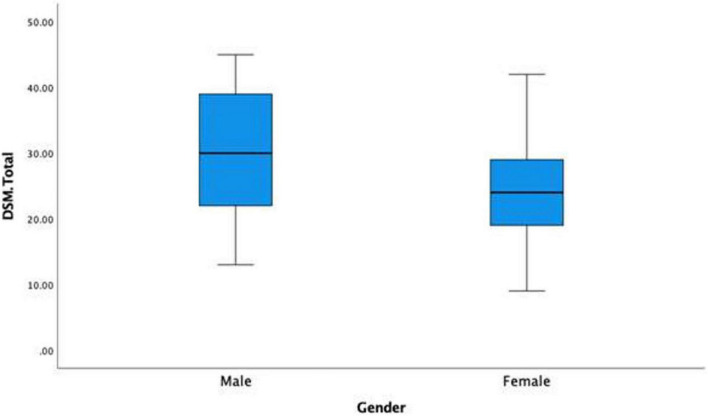
Boxplot of the means and SDs of internet gaming disorder (IGD) scores based on gender.

There was interest in whether there would be a significant gender difference in IGD scores. To this end, an independent samples *t*-test was conducted to answer this question. The results indicate that the Levene’s test was significant (*p* < 0.001) which meant equal variance could not be assumed (see Figure 2 boxplot for heterogeneity of variance) as males had considerably higher variance in their scores. The *t* test for equal variances not assumed was statistically significant, with the male group (*M* = 29.45, *SD* = 9.23) reporting on average 5.11 units higher on IGD scores compared to the female group (*M* = 24.34, *SD* = 7.14), 95% CI [3.04,7.18], *t*(208.19) = 4.87, *p* < 0.001, *d* = 0.63.

A secondary analysis was implemented to analyse those participants who exhibited high IGD scores and therefore met the DSM-5 criteria for IGD (responding *strongly agree* for at least 5 out of 9 symptoms). The descriptive statistics found that the prevalence rate of high IGD (renamed *IGD_Clinical*) scores in the present sample was 6.2% (*n* = 16) (see Table 4).

**TABLE 4:** Means, standard deviations, and *t*-test for equality of means of IGD.Total according to gender.

	Gender	*n*	*M*	*SD*	*t*	*df*	Sig.
IGD	Male	114	29.45	9.23	4.87	208.19	<0.001
	Female	145	24.34	7.14			

Based on this new clinical group (*IGD_Clinical*), an independent samples *t*-test was conducted to evaluate whether there would be a significant difference between respondents in IGD_Clinical (*n* = 16) and non-clinical on Sensation Seeking scores (*n* = 243) (see Table 5). Levene’s test was not significant, therefore equal variances was assumed. The *t* test was significant, with the IGD_Clinical (*M* = 62.25, *SD* = 7.37) reporting on average 13.13 points higher on Sensation Seeking scores (B-SSS), CI 95% [7.56,18.69], than the sub-clinical group (*M* = 49.12, *SD* = 11.14), *t*(257) = 4.64, *p* < 0.001, *d* = 1.2 (see Table 6).

**TABLE 5:** Summary of percentages of IGD_clinical compared to IGD_non-clinical on gender, age, academic, and gaming hours.

	Category	IGD_clinical (*N* = 16) *n* (%)	IGD_non-clinical (*N* = 243) *n* (%)
Gender	Male	13 (81.3)	101 (41.6)
	Female	3 (18.8)	142 (58.4)
Age (years old)*	10	4 (25)	9 (3.7)
	12	2 (12.5)	9 (3.7)
	13	4 (25)	28 (11.5)
	15	6 (37.5)	26 (10.7)
Academic	Elementary	5 (31.3)	71 (29.2)
	Prep-school	7 (43.8)	18 (7.4)
	High school	4 (25)	154 (63.4)
Gaming hours*	1–3 h	2 (12.5)	53 (21.8)
	3–6 h	8 (50)	30 (12.3)
	6–9 h	4 (25)	20 (8.2)
	>9 h	2 (12.5)	24 (9.9)

*Age years in IGD_Subclinical group ranges from 8 to 18 and gaming hours there is <1 h category.

**TABLE 6:** Means, standard deviations, and *t*-test for equality of means of SS scores according to clinical and non-clinical internet gaming disorder (IGD) scores.

	IGD	*n*	*M*	*SD*	*t*	*df*	Sig.
SS	Clinical	16	62.25	7.37	4.64	257	<0.001
	Non-clinical	243	49.12	11.14			

Another research question of interest was whether participants’ grade level significantly affected their scores on SS and IGD. To this end, a one-way MANOVA was conducted to determine the effect of school grades (elementary, prep-, and high-school) on the two dependent variables, SS and IGD. As all underlying assumptions were supported by the data, a MANOVA was conducted. Findings showed that there was a significant effect of education level on SS and IGD levels, *F*(4,510) = 5.15, *p* < 0.001, partial η^2^ = 0.039.

Analysis of the dependent variables individually showed no effect of SS scores as a function school level. However, the IGD scores were statistically significant at a Bonferroni adjusted alpha level of 0.025, *F*(2,256) = 8.75, *p* = <0.001, partial η^2^ = 0.064. This indicated that scores on IGD significantly differed as a function of school grade (see Table 7).

**TABLE 7:** *Post hoc* analysis of univariate analysis of variance (ANOVA) for internet gaming disorder (IGD).

Dependent variable	School grade	*M*	*SD*
SS	Elementary	50.36	11.37
	Prep school	53.00	11.37
	High school	49.24	11.38
IGD	Elementary	29.17	8.26
	Prep school	29.56	8.26
	High school	24.87	8.25

Table 8 shows the descriptive statistics for elementary (*n* = 76), prep school (*n* = 25), and high school (*n* = 158) on each dependent variable.

**TABLE 8:** *Post hoc* analysis of univariate analysis of variance (ANOVA) for internet gaming disorder short scale (IGDSS).

Dependent variable	Hours	*M*	*SD*
B-SSS	<1 h	46.97	10.65
	1–3 h	47.91	10.66
	3–6 h	56.68	10.66
	6–9 h	49.57	10.65
	>9 h	57.77	11.09
IGDSS	<1 h	21.38	6.86
	1–3 h	27.93	6.86
	3–6 h	32.58	5.45
	6–9 h	30.33	6.85
	>9 h	34.77	6.85

*Post hoc* analysis of univariate analysis of variance (ANOVA) for IGD scores was conducted to ascertain which school grades differed significantly on IGD scores. Based on Tukey HSD pairwise comparisons, high school scores were significantly lower than elementary (M difference = −4.30, *p* < 0.001) and prep school (M difference = −4.69, *p* = 0.024). No significant differences were found between elementary and prep school IGDSS scores (see Figure 4).

A further research question of interest was the extent to which the amount of gaming hours would produce significantly different scores on Sensation Seeking and IGD. To this end, a one-way MANOVA was conducted to ascertain levels of IGDSS and B-SSS as a function of the number of hours’ participants played online games. As all underlying assumptions were supported by the data, a MANOVA was conducted. Findings showed that there was a significant effect of number of hours (<1, 1–3, 3–6, 6–9, and >9 h) on scores on IGDSS and B-SSS scores, *F*(8,506) = 17.85, *p* < 0.001, partial η^2^ = 0.220.

Analysis of the dependent variables individually showed significant difference as a function of hours played. B-SSS scores were statistically significant at a Bonferroni adjusted alpha of 0.025, *F*(4,254) = 10.08, *p* < 0.001, partial η^2^ = 0.137. Similarly, IGDSS scores differed significantly at a Bonferroni adjusted alpha of 0.025, *F*(4,254) = 35.52, *p* < 0.001, partial η^2^ = 0.359 (see Table 8).

Table 8 shows the descriptive statistics for <1 h (*n* = 116), 1–3 h (*n* = 55), 3–6 h (*n* = 38), 6–9 h (*n* = 24), and >9 h (*n* = 26) on each dependent variable.

*Post hoc* analysis of univariate ANOVA for IGDSS scores was conducted to ascertain what hour groups differed significantly between each other on gaming disorder scores. Based on Tukey HSD pairwise comparisons, it was found that participants in the <1 h group had significantly lower IGDSS scores compared to 1–3 h (M difference = −6.55, *p* < 0.001), 3–6 h (M difference = −11.20, *p* < 0.001), 6–9 h (M difference = −8.95, *p* < 0.001), and >9 h (M difference = −13.39, *p* < 0.001). Furthermore, 1–3 h group was significantly lower than 3–6 h (M difference = −4.65, *p* = 0.013) and >9 h (M difference = −6.84, *p* < 0.001), but not of 6–9 h. In addition, 3–6 h group was not significantly higher nor lower than 6–9 h and >9 h groups. Finally, >9 h was significantly greater than <1 h and 1–3 h, but not 3–6 and 6–9 h (see Figure 5).

A second *post hoc* analysis of univariate ANOVA for B-SSS scores was carried out to verify if sensation seeking scores differed significantly between hour groups. Based on Tukey HSD pairwise comparisons, it was found that those in the <1 h group showed significantly less B-SSS scores than 3–6 h (*M difference* = −9.72, *p* < 0.001) and >9 h (*M difference* = −10.80, *p* < 0.001), but not 1–3 h nor 6–9 h. Furthermore, 1–3 h was significantly less than 3–6 h (*M difference* = −8.78, *p* = 0.001) and >9 h (*M difference* = −9.86, *p* = 0.001). Finally, the 6–9 h group was not significantly different to any other hour group (see Figure 6).

## Discussion

The present study investigated IGD and associations with sensation seeking in a sample of Arab adolescents. This study is one of a very small number of studies to examine IGD amongst an Egyptian sample. The results showed that there was a significant positive association between SS and IGD; as SS scores increased, IGD severity scores also increased. This is in accordance with past research (35, 36, 50–54). Furthermore, SS scores explained a substantial amount of variance in IGD severity scores. On average, male participants displayed higher SS scores and higher IGD severity scores than female participants, this is in accordance with previous research (55, 56).

**FIGURE 3:**
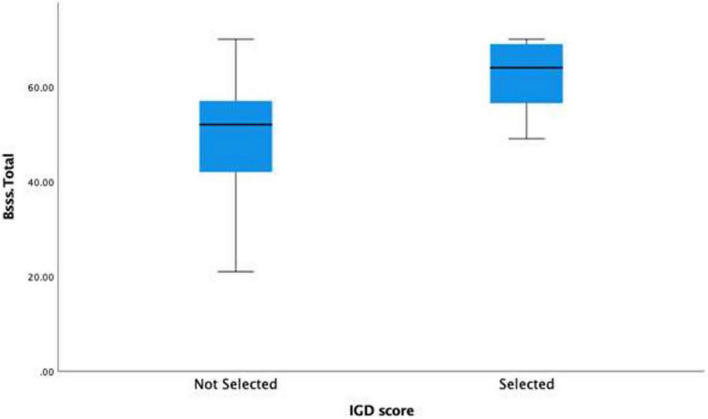
Boxplot of the means and SDs of sensation seeking disorder based on clinical (selected) and non-clinical (not selected) internet gaming disorder (IGD) scores.

The results also revealed that the clinical IGD severity prevalence rate was 6.2%, this was higher than prevalence rates reported in a German sample [3.5%, (56)], and in a Chinese sample [4.3%, (57)]. When comparing the clinical IGD severity and non-clinical IGD severity group, the results showed that the clinical IGD severity group reported higher SS scores than the non-clinical IGD severity group (see Figure 3). Similar findings were reported by previous research studies (36, 37). Furthermore, analysis of time spent online gaming revealed that playing for less than 1 h a day was associated with significantly lower IGD severity scores, playing for more than 3 h was associated with significantly higher IGD severity scores. These findings support previous research that has reported associations between increased time playing and IGD (16, 26, 27, 46).

**FIGURE 4:**
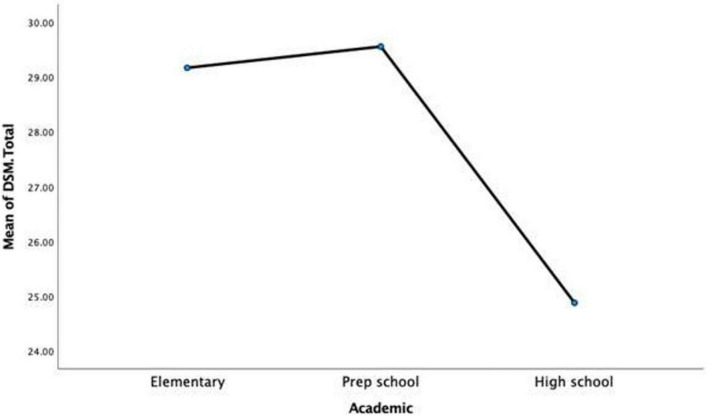
Mean internet gaming disorder short scale (IGDSS) scores on elementary, prep school, and high school grades.

**FIGURE 5:**
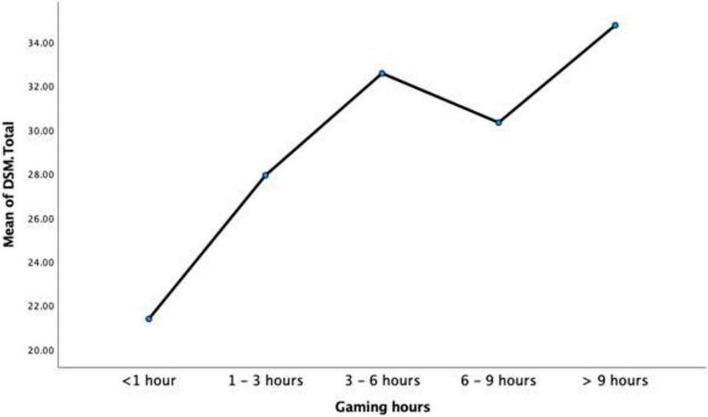
Mean internet gaming disorder short scale (IGDSS) scores as a function of gaming hours.

**FIGURE 6:**
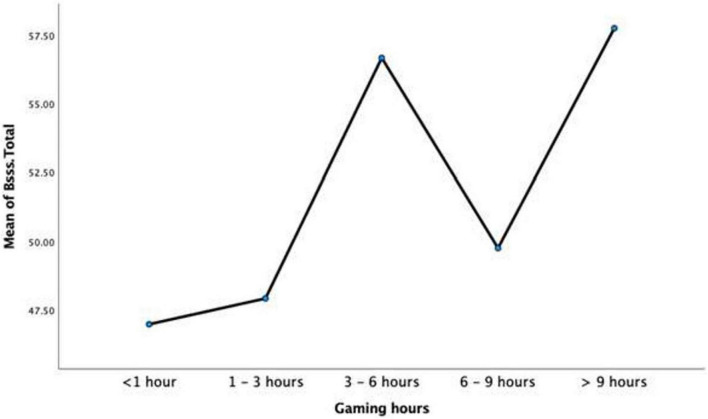
Mean brief sensation seeking scale (B-SSS) scores as a function of gaming hours.

In regards to SS, playing for less than an hour or between 1 and 3 h was associated with significantly lower SS scores. This finding contributes to the research literature by revealing that lower amounts of time gaming can be a buffer against the need to use online gaming as a sensation seeking experience. It can be speculated that the desire to experience arousing feelings may be associated with increased gaming time. The cognitive-behavioural model of IGD (28) can be applied here, gamers may begin to engage in gaming for reward-seeking and stress-reduction purposes, then later make a decision to stop playing after weighing up the consequences of excessive playing. Gamers may be implementing behavioural control mechanisms, further research studies that examine the decision making processes of gamers is needed.

While this study provides important insights into the online gaming behaviour of Arab adolescents and sensation seeking, the study has some limitations. The recruitment of a non-clinical sample meant that the generalisability of the findings was reduced. The use of self-report methods meant that the effects of recall bias could not be excluded. Future research should attempt to tackle these limitations. Furthermore, future research could investigate family relationships as a potential factor associated with IGD, research has reported that poor quality parent-child relationships are associated with increased IGD severity (58). Longitudinal and intervention studies are needed to help improve understanding and provide treatment.

The present study is an important step towards understanding IGD and SS. The study found associations between SS and IGD, higher SS scores were reported among the clinical IGD severity group, and male participants reported higher SS and IGD severity scores. The study findings provided important insights into an under-researched culture and will have implications for the development of interventions.

## Data Availability Statement

The original contributions presented in this study are included in the article/Supplementary Material, further inquiries can be directed to the corresponding author.

## Author Contributions

MH came up with the idea for the study. ZH helped come up with the plan for the study. EA did the testing and data collection. MH analyzed and interpreted the data under the direction of AA and EA wrote the manuscript. AA and ZH made important changes and gave the reviewers’ corrections. All authors agreed that the final version of the manuscript was ready to be sent out and agreed that they all made equal contributions to this manuscript.

## Conflict of Interest

The authors declare that the research was conducted in the absence of any commercial or financial relationships that could be construed as a potential conflict of interest.

## Publisher’s Note

All claims expressed in this article are solely those of the authors and do not necessarily represent those of their affiliated organizations, or those of the publisher, the editors and the reviewers. Any product that may be evaluated in this article, or claim that may be made by its manufacturer, is not guaranteed or endorsed by the publisher.
