# Supplementary material for: The Association Between Internet Gaming Disorder and Sensation Seeking Among Arab Adolescents
Source: Front Psychiatry. 2022 Jul 15;13:905553. doi: 10.3389/fpsyt.2022.905553 (PMC9334920; doi:10.3389/fpsyt.2022.905553)
Supplement: Supplementary file 1 [file Table_1.DOCX]

Table 1. *Summary of correlations between Zscore Bsss.Total and Zscore DSM.Total*

| Correlations | | | |
| --- | --- | --- | --- |
|  |  | Zscore(Bss.Total) | Zscore (DSM.Total) |
| Zscore(Bss.Total) | Pearson Correlation | 1 | .587** |
|  | Sig.(2-tailed) |  | <.001 |
|  | *N* | 259 | 259 |
| Zscore(DSM.Total) | Pearson Correlation | .587** | 1 |
|  | Sig. (2-tailed) | <.001 |  |
|  | *N* | 259 | 259 |

**. Correlation is significant at 0.01 level (2-tailed)

Table 2. *Means, Standard Deviations and t-test for Equality of means of Bsss.Total according to gender*

|  | Gender | *n* | *M* | *SD* | *t* | *df* | Sig. |
| --- | --- | --- | --- | --- | --- | --- | --- |
| Bsss.Total | Male | 114 | 51.65 | 11.60 | 2.17 | 257 | 0.031 |
|  | Female | 145 | 48.59 | 11.06 |  |  |  |

Table 3. *Means, Standard Deviations and t-test for Equality of means of DSM. Total according to gender*

|  | Gender | *n* | *M* | *SD* | *t* | *df* | Sig. |
| --- | --- | --- | --- | --- | --- | --- | --- |
| DSM.Total | Male | 114 | 29.45 | 9.23 | 4.87 | 208.19 | <.001 |
|  | Female | 145 | 24.34 | 7.14 |  |  |  |

Table 4. *Summary of percentages of IGD_Clinical compared to IGD_non-clinical on Gender, Age, Academic and Gaming Hours.*

|  | Category | IGD_Clinical (*N*=16)  *n*(%) | IGD_non-clinical (*N*=243) *n*(%) |
| --- | --- | --- | --- |
| Gender | Male | 13(81.3) | 101(41.6) |
|  | Female | 3(18.8) | 142(58.4) |
| Age (years old) * | 10 | 4(25) | 9(3.7) |
|  | 12 | 2(12.5) | 9(3.7) |
|  | 13 | 4(25) | 28(11.5) |
|  | 15 | 6(37.5) | 26(10.7) |
| Academic | Elementary | 5(31.3) | 71(29.2) |
|  | Prep-School | 7(43.8) | 18(7.4) |
|  | High School | 4(25) | 154(63.4) |
| Gaming Hours* | 1-3 hours | 2 (12.5) | 53(21.8) |
|  | 3-6 hours | 8(50) | 30(12.3) |
|  | 6-9 hours | 4(25) | 20(8.2) |
|  | >9 hours | 2(12.5) | 24(9.9) |

**Note*. Age years in IGD_Subclinical group ranges from 8-18 and Gaming Hours there is <1hour category.

Table 5. *Means, Standard Deviations and t-test for Equality of means of B-SSS_Total according to Clinical and Non-clinical IGD scores.*

|  | IGD | *n* | *M* | *SD* | *t* | *df* | Sig. |
| --- | --- | --- | --- | --- | --- | --- | --- |
| B-SSS.Total | Clinical | 16 | 62.25 | 7.37 | 4.64 | 257 | <.001 |
|  | Non-Clinical | 243 | 49.12 | 11.14 |  |  |  |

Table 6*. Post-hoc analysis of univariate ANOVA for IGD*

| *Dependent variable* | *School Grade* | *M* | *SD* |
| --- | --- | --- | --- |
| *B-SSS* | *Elementary* | *50.36* | *11.37* |
|  | *Prep School* | *53.00* | *11.37* |
|  | *High School* | *49.24* | *11.38* |
| *IGDSS* | *Elementary* | *29.17* | *8.26* |
|  | *Prep School* | *29.56* | *8.26* |
|  | *High School* | *24.87* | *8.25* |

Table7. *Post-hoc analysis of univariate ANOVA for IGDSS*

| *Dependent variable* | *Hours* | *M* | *SD* |
| --- | --- | --- | --- |
| *B-SSS* | *< 1 hour* | *46.97* | *10.65* |
|  | *1-3 hours* | *47.91* | *10.66* |
|  | *3-6 hours* | *56.68* | *10.66* |
|  | *6-9 hours* | *49.57* | *10.65* |
|  | *>9 hours* | *57.77* | *11.09* |
| *IGDSS* | *< 1 hour* | *21.38* | *6.86* |
|  | *1-3 hours* | *27.93* | *6.86* |
|  | *3-6 hours* | *32.58* | *5.45* |
|  | *6-9 hours* | *30.33* | *6.85* |
|  | *>9 hours* | *34.77* | *6.85* |
